# Diseased, differentiated and difficult: Strategies for improved engineering of *in vitro* neurological systems

**DOI:** 10.3389/fncel.2022.962103

**Published:** 2022-09-27

**Authors:** Nicholas Elder, Faranak Fattahi, Todd C. McDevitt, Lyandysha V. Zholudeva

**Affiliations:** ^1^Department of Cellular and Molecular Pharmacology, University of California, San Francisco, San Francisco, CA, United States; ^2^Eli and Edythe Broad Center of Regeneration Medicine and Stem Cell Research, University of California, San Francisco, San Francisco, CA, United States; ^3^Gladstone Institutes, San Francisco, CA, United States; ^4^Department of Bioengineering and Therapeutic Sciences, University of California, San Francisco, San Francisco, CA, United States; ^5^Sana Biotechnology, Inc., South San Francisco, CA, United States

**Keywords:** stem cells, cellular engineering, directed neurons, induced neurons, micro RNA, transcription factor complexes

## Abstract

The rapidly growing field of cellular engineering is enabling scientists to more effectively create *in vitro* models of disease and develop specific cell types that can be used to repair damaged tissue. In particular, the engineering of neurons and other components of the nervous system is at the forefront of this field. The methods used to engineer neural cells can be largely divided into systems that undergo directed differentiation through exogenous stimulation (i.e., *via* small molecules, arguably following developmental pathways) and those that undergo induced differentiation *via* protein overexpression (i.e., genetically induced and activated; arguably bypassing developmental pathways). Here, we highlight the differences between directed differentiation and induced differentiation strategies, how they can complement one another to generate specific cell phenotypes, and impacts of each strategy on downstream applications. Continued research in this nascent field will lead to the development of improved models of neurological circuits and novel treatments for those living with neurological injury and disease.

## Introduction

Proper function of the nervous system depends on precise orchestration of numerous neural and non-neural (glial, vascular, etc.) cell types to create complex networks. While there is some neuroplastic potential, damage to mature neural networks due to injury or disease is often irreversible. Neurons are especially susceptible to permanent loss of function when these networks are compromised in conditions ranging from acute traumatic injury to chronic neurodegeneration (Kwon et al., 2004; Ahuja et al., 2017). Abnormal development, loss of circuit relay, or selective degeneration of neurons can result in life-long and debilitating outcomes, with limited chance of neural regeneration or spontaneous replacement. This has led to a growing interest in the promise of cell-transplantation therapies for neural diseases, including in engineering relevant cell types *in vitro* to replace those lost in specific disease or injury settings (Assinck et al., 2017; Zholudeva and Lane, 2019; Fischer et al., 2020; Piao et al., 2021).

Here, we highlight the expanding toolbox of techniques used to derive neurons from pluripotent stem cells (PSCs), and describe their utility in research and preclinical settings for disease modeling and nervous system repair. We propose that combining aspects of directed and induced differentiations could improve cell type specificity, purity, scalability, and modularity in downstream applications. Specifically, we will discuss how different methods of morphogen and chemical stimulation, transcription factor overexpression, transcription factor cooperation, microRNA regulation, and fate “blocking” can be utilized to generate defined populations of neurons.

## Directed versus induced differentiation

**FIGURE 1:**
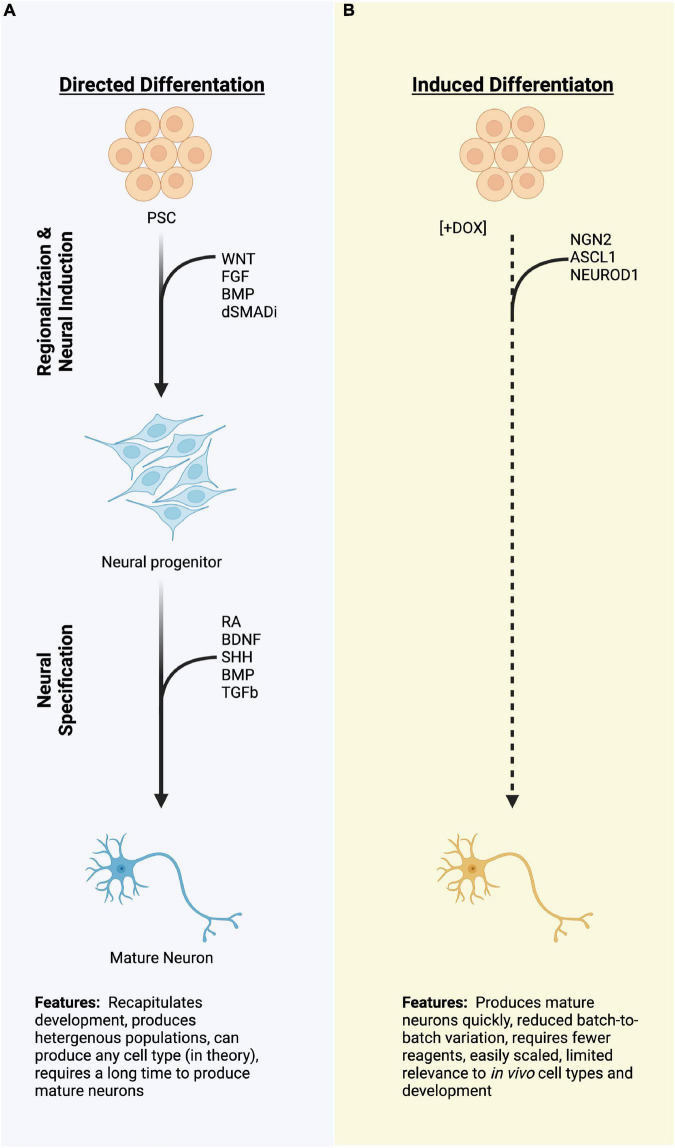
A schematic diagram of two strategies for differentiating pluripotent stem cells (PSCs) to neurons. **(A)** Directed differentiation employing exogenous manipulation of common signaling pathways to produce neural progenitors and neurons. **(B)** Induced differentiation from PSCs using overexpression of proneural transcription factors which quickly produce a neuronal molecular fate. Dashed lines represent rapid processes due to induced TF expression as compared to the directed differentiation, which typically take a longer time. PSCs, pluripotent stem cells; TFs, transcription factors; miRNA; (Created with BioRender.com).

The generation of neurons from human PSCs *in vitro* largely centers around two approaches: directed differentiation and induced differentiation. Directed differentiation recapitulates the developmental progression from stem cells to neurons by employing exogenous factors, such as recombinant proteins or small molecules, to stimulate or antagonize signaling pathways known to be important during development of the cell types of interest (Figure 1A). For example, dual inhibition of BMP and TGFb signaling is necessary to specify the anterior neural plate during gastrulation (Camus et al., 2006; Smith et al., 2008; Muñoz-Sanjuán and Brivanlou, 2022). This can be recapitulated *in vitro* by the addition of recombinant proteins, such as Noggin, or more commonly by small molecule compounds which antagonize the intracellular relay of TGF and BMP signals (Smith et al., 2008; Chambers et al., 2009, 2012). The cells are sequentially transitioned through increasingly restricted progenitor populations to achieve target neural identity, and protocols utilizing this strategy have been described for numerous neural types (Wichterle et al., 2002; Li et al., 2005; Kriks et al., 2011; Shi et al., 2012; Fattahi et al., 2016; Butts et al., 2017). Because cells are transitioned *via* exogenous stimulation, directed differentiations rely on an intrinsic rate of developmental progression, can take weeks to months to produce post-mitotic target cell populations, and even longer for those cells to display appropriate physiological characteristics. These long differentiations also typically result in heterogeneous populations of neurons from target nervous system regions (Merkle et al., 2015; Butts et al., 2017; Sloan et al., 2018). This heterogeneity can be useful, for instance when studying developmental determinants which promote one neural fate over the other, especially when the decision points of such fate decisions are unknown (Hoang et al., 2018; Bhaduri et al., 2020). Further, when cultured in three-dimensional organoids, mixed populations of progenitors and neurons from directed differentiations can give rise to highly patterned tissues that closely resemble *in vivo* development, such as the layers of the cortex or the folding of the optic cup (Eiraku et al., 2011; Kadoshima et al., 2013; Lancaster et al., 2013), enabling investigation of complex aspects of nervous system development (Qian et al., 2016; Tang et al., 2016; Bershteyn et al., 2017; Pollen et al., 2019; Birey et al., 2022). However, the balance of specific neural populations can shift dramatically batch-to-batch within a single cell line and between cell lines, decreasing the translational potential of such strategies for targeted therapeutic applications (Volpato et al., 2018). This is not to say that it is impossible—indeed, PSC-derived dopaminergic neurons are currently entering clinical trials to treat Parkinson’s disease (Doi et al., 2020; Schweitzer et al., 2020; Kim et al., 2021; Piao et al., 2021). Directed differentiation from stem cells to neurons using exogenous factors remains a standard approach across the field.

By contrast, direct induction uses genetic engineering techniques to “turn-on” expression of neurogenic factors to generate a post-mitotic neural fate directly from stem cells (Figure 1B). This process bypasses or drastically abbreviates developmental progression, and shortens the amount of time that it takes to generate neurons, with morphological changes occurring within 72 h in human cells and spontaneous electrical activity observable in as little as 4 weeks (Zhang et al., 2013; Busskamp et al., 2014; Wang et al., 2017). Not only is the speed of the induction beneficial, but induced neuron populations tend to be more homogenous than those produced by directed differentiation, both within and between batches (Zhang et al., 2013). By combining induced expression of pro-neural transcription factors, usually bHLH family proteins, with cell type-specific transcription factors, induced neurons can be further directed to adopt highly specific cell fates. Combinations of transcription factors (TFs) have been used to produce dopaminergic neurons, sensory neurons, motor neurons, and cortical GABAergic neurons, among others (Goto et al., 2017; Yang et al., 2017; Azimi et al., 2018; Nickolls et al., 2020). Such targeted strategies allow for screening of disease phenotypes (e.g., disease-in-a-dish), high-throughput drug screens, and could potentially serve as a cell source for cell replacement therapies (Wang et al., 2017; Feliciano et al., 2021). However, induced approaches exist for very few of the vast diversity of neuron types within the body.

## Source of cells

The starting cellular material is an important consideration when producing neurons for disease modeling or potential therapeutic applications. This review is focused on stem cells as the starting source for both directed and induced differentiations to allow for comparison between these methods and to contrast with *in vivo* development. Early experiments used somatic cells, typically astrocytes or fibroblasts, as a starting point to screen for factors with direct reprogramming or transdifferentiation (i.e., across germ lineage) neurogenic potential, which were subsequently successfully applied to stem cells (Berninger et al., 2007; Vierbuchen et al., 2010; Bocchi et al., 2022). Furthermore, neurons induced directly from fibroblasts retain epigenetic markers of aging, and thus can be useful for screening age-related phenotypes that are difficult to recapitulate in stem cell-derived neurons (Mertens et al., 2015; Yang et al., 2015). Thus, fibroblasts and other somatic cell types, such as glia, are a useful cell source for screening and age-associated disease modeling.

Pluripotent stem cells, by contrast, can be more flexibly programmed or directed to desired cell types. The discovery of induced pluripotent stem cells (iPSCs) drastically improved stem-cell based disease models (Takahashi and Yamanaka, 2006; Takahashi et al., 2007; Yu et al., 2007). iPSC technology has allowed research to generate cell lines from individuals with clinically confirmed illness, and thus study disease phenotypes in the genetic contexts known to present disease phenotypes. iPSC lines from patients can also be matched with “unaffected” cell lines derived from siblings or parents. Alternatively, scientists can generate a defined mutation or set of mutations in a “wild-type” cell line. However, due a re-setting of the epigenetic clock during reprogramming, or soon after fertilization in the case of embryonic stem cells (ESCs), stem cells lack many markers of age, and thus make for imperfect models of age-related disease (Marion et al., 2009; Suhr et al., 2010; Kerepesi et al., 2021; Simpson et al., 2021).

Stem cell lines can also possess lineage biases, both between genetically unique donors and between uniquely isolated clones from the same donor, which can manifest as a “preference” to differentiate to certain germ lineages over others (Osafune et al., 2008; Boulting et al., 2011; Strano et al., 2020). While this poses less of a hurdle in induced differentiations, which employ strong over-expression of transcription factors, it can shift the outcome of directed differentiations. In addition to innate lineage bias, PSC lines can also exhibit differing sensitivities to morphogens. The most common example of this is sensitivity to WNT signaling. The concentration of CHIR99021, a GSK3 inhibitor and agonist of the WNT pathway frequently used in neural differentiations, has to be routinely titrated for each cell line to achieve similar results (Ashton et al., 2015; Strano et al., 2020; Libby et al., 2021).

Lastly, when considering the production of neurons for therapeutic applications, cell lines must be produced and maintained under current good manufacturing practices (cGMPs), including use of defined, xeno-free culture reagents, testing for viral or bacterial contaminants, and confirming normal karyotypes. Both ESC and iPSC lines have been produced under cGMP conditions and are commercially available. Although there are many considerations to working with stem cells, their ability to be differentiated through both directed and induced methods into neurons has made them an invaluable tool to study development and model disease, and has opened new avenues for personalized medicine and potential cell replacement therapies.

## Regionalization—the foundation of cell identity

One of the earliest processes in post-gastrulation development is the axial rostro-caudal (head–to-tail) specification of the nervous system. In the gastrula, the anterior neural plate gives rise to the forebrain, while caudalizing signals originating from the node at the posterior of the embryo and, later, anterior somites are necessary to induce hindbrain identity, as well as to specify a pool of axial stem cells called neuromesodermal progenitors (NMPs) (Tzouanacou et al., 2009; Wymeersch et al., 2016; Metzis et al., 2018; Frank and Sela-Donenfeld, 2019). NMPs are maintained around the node-streak border during axial elongation, and proliferate to give rise to both spinal cord progenitors and presomitic mesoderm. The allocation of different progenitors to different regions of the rostrocaudal axis at such an early time in development underscores how critical this step is in preventing unwanted cell types from arising in the wrong location. Even a transcription factor as ubiquitous in the nervous system as SOX2 shows differential chromatin binding in the developing embryo depending on the region-specific cofactors available (Hagey et al., 2016). For these reasons, regionalizing steps are critically important in any differentiation protocol, as this process sets up the chromatin landscape upon which downstream, cell-type-specific TFs act, while also limiting the number of potential cell fates.

Throughout the nervous system, regional identity is underwritten and reinforced by the expression of TFs containing highly evolutionarily conserved homeobox domains. Homeobox gene families important for forebrain and midbrain development include LHX, OTX, SIX, and DLX (some of which are also used in induced differentiation strategies). However, among the most well-studied regionally specific homeobox proteins are the HOX and CDX gene families, which are expressed at hindbrain levels and below. Consisting of 39 genes across four unique clusters in mice and humans, HOX proteins demarcate functionally and anatomically distinct regions within the hindbrain and spinal cord (Dasen et al., 2005; Philippidou and Dasen, 2013; Deschamps and Duboule, 2017). HOX genes play a central role in hindbrain rhombomere formation, with deletion or misexpression of anterior HOX genes resulting in loss or expansion of unique rhombomere domains (Frank and Sela-Donenfeld, 2019; Parker and Krumlauf, 2020). In the spinal cord, HOX genes help direct motoneuron identity, with distinct motor pools and columns generated at limb and trunk-innervating levels (Dasen et al., 2005; Dasen and Jessell, 2009; Jung et al., 2014). Homeobox-containing genes are frequently some of the earliest genes whose expression is differentially segregated into different regions of the nervous system, and they act as downstream effectors of regionalization.

Fortunately, extensive study in embryology has uncovered the major signaling determinants of regionalization, allowing for these processes to be recapitulated *in vitro*. For example, removal of stem cell maintenance signals and/or dual inhibition of TGFb and BMP pathways (also known as dual SMAD inhibition) is sufficient to produce SOX2 + /PAX6 + forebrain neural stem cells from PSCs (Smith et al., 2008; Chambers et al., 2009, 2012; Lippmann et al., 2014). To obtain neurons of hindbrain or high spinal cord identity, treatment of stem cells with either retinoic acid or low levels of Wnt is sufficient to upregulate rostral HOX paralogs 1–6, usually in conjunction with or even after neural induction with dual SMAD inhibition (Butts et al., 2017; Valiulahi et al., 2021). However, protocols to generate cells representing the most caudal segments of the spinal cord, including lumbar and sacral regions expressing HOX paralogs 10–13, were lacking until researchers learned to transition stem cells through a NMP state capable of upregulating these genes (Lippmann et al., 2015; Kumamaru et al., 2018; Wind et al., 2021). While cooperative FGF and Wnt activity is necessary to induce cervical and thoracic HOX genes (HOX7-9), caudalization of the cellular identity toward the lumbar level requires further stimulation by GDF11 to express HOX 10 and beyond (Mazzoni et al., 2013b; Lippmann et al., 2015). To date, most protocols to generate neurons of spinal identity achieve only rostral HOX gene expression, encompassing only cervical regions of the spinal cord, which innervate the forelimbs. Apart from the central nervous system (CNS), neurons of the peripheral nervous system (PNS), including sensory and enteric neurons, transition through a neural crest lineage during development. Therefore, differentiation protocols for these populations incorporate a period of neural crest induction, requiring Wnt and BMP, in conjunction with dual SMAD inhibition for neural induction (Denham et al., 2015; Fattahi et al., 2016; Alshawaf et al., 2018).

Emerging evidence points to the action of HOX genes in regulating and maintaining post-mitotic and post-natal neural identity as well (Feng et al., 2021), demonstrating that HOX expression is not only necessary for developmental patterning, but also strongly influences circuit connectivity and mature neural function. Osseward et al. (2021) reported that a k-means clustering approach to divide lumbar neurons from newborn mice revealed that the first major division between neurons did not resolve into neurotransmitter identity, as expected, but instead divided sensory laminae I-III from HOXC10 expressing neurons in laminae IV-X involved in motor control and proprioception (Osseward et al., 2021). Though not a direct tie to neural function, this evidence points to HOX gene function throughout spinal motor networks, not just in motor neurons themselves, as has been well-described.

Notably, genetic signatures of regional identity can persist through *ex vivo* culture across many passages and even through direct reprogramming (Kelly et al., 2009). Regional signatures are also maintained in expanded pools of neural stem cells, and can either help to reinforce cell identity or prevent adoption of cell fates that are not matched to the region of the starting cellular material (Kumamaru et al., 2018). For example, neurons induced from cortical gray matter or spinal cord-derived astrocytes retained markers of regional identity, particularly in the HOX genes expressed, producing neurons with more spinal or more cortical-like identities, respectively (Kempf et al., 2021). Similarly, neural induction *via* NGN2 overexpression yielded a mixed population of cells with CNS and PNS characteristics that were resolved by short regional patterning steps prior to NGN2 induction (Chen et al., 2020; Lin et al., 2021). Dual SMAD inhibition was sufficient to specify cortical-like populations, whereas treatment with a TGFb inhibitor and Wnt agonist prior to induction specified hindbrain-like populations. Further, overexpression of specific HOX TFs in *in vitro* differentiated motor neurons shows that individual HOX genes can bind thousands of different regions, changing the chromatin landscape and the resulting motoneuron subtype under otherwise uniform induction conditions (Bulajić et al., 2020). It remains unknown, however, the extent to which expression of individual HOX genes can recapitulate the regulatory landscape that is produced by sequential activation of all of the HOX clusters simultaneously, as happens in development. Thus, regionalization steps are complementary to TF-driven neural induction, increasing the specificity of the resulting populations. Regionalization using readily available potent small molecule modulators or recombinant proteins can be a fast process during the relative timeline of neuron differentiation and maturation. It is also a critical step that informs all downstream processes of neuronal subtype specification.

## Neural induction

Following regionalization, which may require maintaining a multipotent progenitor pool such as neural crest cells or NMPs, cells must be further coaxed toward a neural lineage. During development, an ever-changing milieu of morphogenic gradients, metabolites, and extracellular environments direct transcriptional and epigenetic changes that influence downstream cell fate. In order to faithfully recapitulate this process *in vitro*, cells must be sequentially passed through distinct developmental states that require time to adapt and to further progress. Thus, directed differentiations tend to rely on prior *in vivo* studies that have determined the major underlying signaling cues and developmental processes that give rise to target populations (Sagner and Briscoe, 2019). In induced differentiations, many of these developmental steps are skipped over by the expression of one or more factors that can efficiently direct cell fate in the absence of otherwise necessary extrinsic cues.

Some of the most commonly utilized induction systems, and indeed the simplest, rely on over-expression of single proneural TFs. These TFs, often members of the bHLH family (including ASCL1, NGN2, NGN1, NEUROD1) are sufficient to induce a neural fate on their own (Flitsch et al., 2020; Bocchi et al., 2022; Hulme et al., 2022). They can act as pioneer factors, binding to and opening regions of chromatin that are normally silent in the base state, and their potency is perhaps amplified by the fact that they can upregulate the endogenous expression of other proneural TFs (Busskamp et al., 2014). Within a short timeframe, neurogenic TFs upregulate a pan-neural transcriptional program, which includes genes for axon and dendrite morphogenesis and synaptic transmission. For example, while initially activating different networks, independent induction of either ASCL1 or NGN2 produce largely similar populations of neurons from spinal cord glia, though ASCL1 has also demonstrated an increased ability to activate inhibitory identity in other models (Yang et al., 2017; Kempf et al., 2021). NGN2 has proven to be especially potent in producing induced neurons, leading to the creation of a human stem cell line harboring a dox-inducible NGN2 cassette that can readily be used to generate large numbers of neurons for screening purposes (Zhang et al., 2013; Wang et al., 2017; Fernandopulle et al., 2018; Tian et al., 2019). While these screens are highly informative, they may miss cell-type specific differences, such as those that make motor neurons or dopaminergic neurons susceptible in ALS and Parkinson’s disease contexts, respectively. In order to study cell type-specific effects, one must either (1) isolate primary cells, (2) follow or develop directed differentiation protocols, or (3) engineer mechanisms to direct the identity of the resulting neural populations.

## Introducing specificity through transcription factor interactions

Because directed differentiations closely recapitulate developmental processes and morphogenic cues, the resulting neural progenitors simultaneously express many TFs necessary for neuron fate specification. By contrast, single-factor induced neurons appear to adopt an excitatory glutamatergic identity most similar in expression pattern to cortical neurons (Zhang et al., 2013; Busskamp et al., 2014). In order to use an induced approach to generate specific neuron sub-types for disease modeling or pre-clinical study, cell type-specific TFs with a known role in the desired cell fate are frequently expressed alongside neurogenic TFs. The number of co-expressed proteins can range from one additional TF to several, depending on the desired cell type. If well-established unique markers exist for a population of interest, screens can be employed to agnostically determine the best combination of TFs to induce. Screens can range in complexity, from screens containing all annotated TFs, to targeted screens of a short-list of likely candidates (Teratani-Ota et al., 2016; Guo and Morris, 2017; Yang et al., 2017; Ng et al., 2021).

However, the co-expression of several TFs should come with considerations of their cooperation, potential cross-repression, timing of expression during development, and duration of induction (Bocchi et al., 2022; Hulme et al., 2022). In tissues with highly conserved and well-described genetics, such as the spinal cord, *in vivo* studies have identified factors that are both necessary and sufficient to induce specific cell fates when ectopically over-expressed. For example, spinal motor neurons (MNs) specifically degenerate in ALS, and homogenous populations of MNs are desired for *in vitro* studies. Developmental studies in chick and mice identified LHX3 as an overlapping marking of the adjacent progenitor domains of spinal V2a interneurons and MNs, while ISL1 was necessary for MN development and expressed solely in the MN domain. Subsequent research found that LHX3, ISL1 and third protein, NLI, form a hexameric complex in a 2:2:2 ratio that potently activates a MN transcriptional program (Tanabe et al., 1998; Thaler et al., 2002; Song et al., 2009; Lee et al., 2012; Erb et al., 2017). By contrast, LHX3 and NLI, in the absence of ISL1, form a tetrameric complex, again in equimolar ratios, that promotes adoption of the V2a fate. Ectopic expression in the dorsal spinal cord of LHX3 alone produces CHX10 + V2a neurons, while co-expression of LHX3 and ILS1 produces HB9 + MNs (Thaler et al., 2002; Song et al., 2009). Knowledge of the specific TF complexes that are sufficient to induce these fates led to the development of mouse and human cell lines that utilize doxycycline-induced expression of LHX3 and ISL1 in equimolar ratios alongside the neurogenic TF NGN2, and which rapidly and homogenously produce MNs in a short time frame (Mazzoni et al., 2013a; Fernandopulle et al., 2018).

**FIGURE 2:**
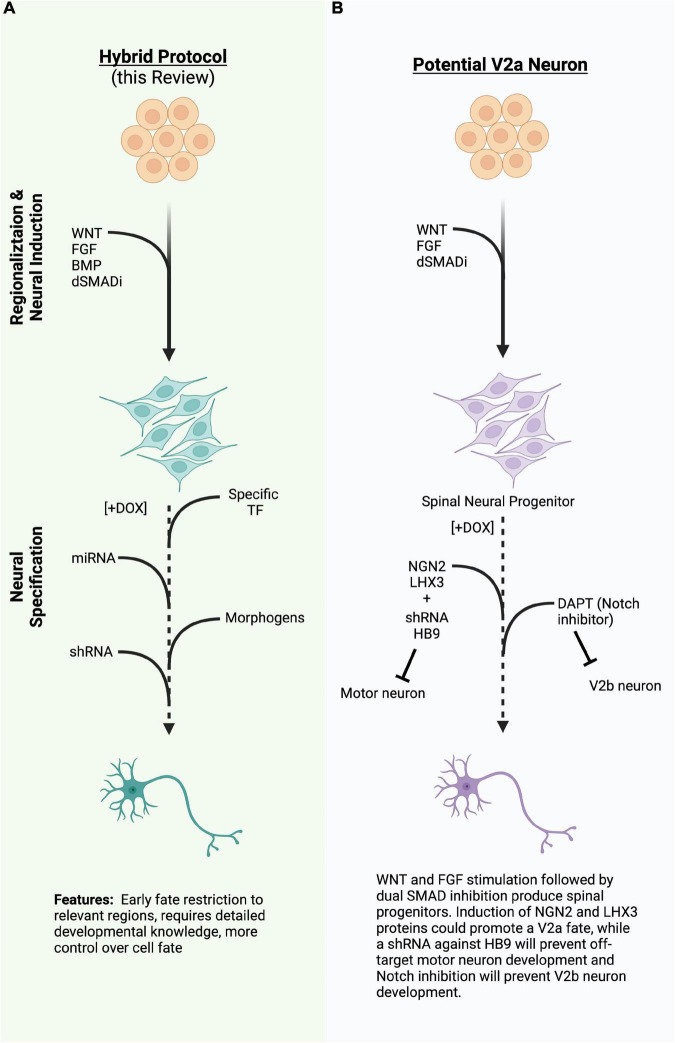
**(A)** A diagram of a hybrid differentiation paradigm employing the same regionalizing steps as directed differentiation while inducing specific transcription factors (TFs), miRNA, or shRNA to quickly induce or refine cell type during neural specification. **(B)** An example of a potential way to generate V2a neurons using the hybrid approach in 2A. TFs, transcription factors; miRNA, micro ribonucleic acid; shRNA, short hairpin ribonucleic acid; (Created with BioRender.com).

Other cooperative systems are known to drive specific spinal cord neuron populations, but have not yet been engineered into induced differentiation systems. For instance, V2a neurons, which have a role in the spinal motor circuitry and central pattern generator (CPG) networks, and degenerate alongside the MN population in ALS (Crone et al., 2012; Azim et al., 2014; Bubnys et al., 2019; Salamatina et al., 2020), could potentially be generated by induced expression of NGN2 and LHX3 in the absence of ISL1 (Figure 2B). Since NLI is abundantly expressed in the developing nervous system, the LHX3:NLI tetrameric complex could be readily assembled. Inhibitory V2b neurons share a common progenitor with V2a neurons, but differentiate toward a GATA2/3 + GABAergic fate due to activated Notch signaling, while V2a neurons require Notch inhibition (Peng et al., 2007). A direct target of Notch activation is the factor SCL, which forms a complex with the LIM competitor LMO4 and NLI (Peng et al., 2007; Joshi et al., 2009). This V2b complex activates and incorporates GATA2, as well as activating GATA3 and GABAergic genes. One could then hypothesize that an inducible cell line containing a proneural factor, such as NGN2 or ASCL1, co-expressed with SCL and LMO4, would produce V2b spinal inhibitory interneurons, though this has yet to be demonstrated. Undoubtedly, many other cooperative TF complexes could be reconstituted in induced systems to promote specific cell fates.

## microRNA-mediated repression as a means to increase cell-type specificity

Another method to direct neural development and fate is through the use of micro-RNA (miRNA). Consisting of approximately 22 nucleotides, miRNAs are highly conserved and abundant in the human genome. Processed from double stranded RNA by Dicer and later bound by the RISC complex, miRNAs bind mRNAs and repress their translation (Bartel, 2004). Similar to how single TFs can activate or repress genes throughout the genome, miRNAs are capable of regulating hundreds of genes simultaneously, biasing cells between states (progenitor to postmitotic neuron) or fates. For example, neuron-specific miR-9/9* functions to balance the neural progenitor and terminal differentiation states by inhibiting Notch activity (Roese-Koerner et al., 2016). Interestingly, miR-9/9* is also a target of Notch, creating a regulatory loop wherein Notch slowly activates its own inhibitor to ensure eventual cell cycle exit and terminal differentiation. MiR-9/9* and miR-124, another neuron specific miRNA, have been used in studies to induce neural fates and facilitate neural maturation (Yoo et al., 2011).

Other miRNAs help to refine cell fate by facilitating boundary formation during development. For example, in the absence of Dicer, Olig2, a TF that helps specify MNs, is expressed more broadly than expected in the developing embryo (Chen et al., 2011). Further work identified miR-17-3p as a miRNA that was upregulated by IRX3, a reciprocal inhibitor of OLIG2 during spinal cord development, and that miR-17-3p targeted the 3’UTR of OLIG2 to spatially restrict its expression. Interestingly, the miRNA cluster that contains miR-17-3p, miR-17∼92, was found to govern postmitotic survival of limb-innervating MNs *via* PTEN regulation (Tung et al., 2015). miRNAs can also regulate boundary formation and positional identity by acting on HOX genes. For example, boundary formation between Hoxa5 and Hoxc8 in the brachial spinal cord was found to rely on miR-27 (Li et al., 2017), while other miRNAs embedded within the HOX clusters themselves contribute to the posterior dominance of HOX genes (Mansfield and McGlinn, 2012). In all, these studies show that miRNA possesses strong regulatory activity *in vivo*, and can have an outsized influence on neuron maturity, fate, and positional identity.

Many methods to derive specific populations of neurons focus on positive enforcement of the desired fate, either through morphogen stimulation or expression of specific transcription factors. However, blocking undesired phenotypes could serve as an alternative route to increase specificity. Short-hairpin RNAs (shRNAs) are processed by the same endogenous machinery as miRNAs, resulting in repression of target transcripts. In both induced and directed systems, shRNAs against specific TFs or miRNAs could serve to canalize identity by blocking alternative routes at the same time as cells receive signals to promote the identity of interest. Indeed, in many cell lineages, loss of key genes that specify one cell fate can lead to the transformation to another (Arlotta and Hobert, 2015).

## Caveats of induced systems

To this point, we have considered *what* to induce, but attention should also be paid to intracellular stresses associated with induction and the method used to express genes of interest. Ectopic overexpression of potent neurogenic genes, such as NGN2, induces a myriad of changes in a short time-span, including transcriptional, epigenetic, morphogenic, and metabolic changes as cells take a fast-track route to a neural identity (Gascón et al., 2016; Ordreau et al., 2021; Russo et al., 2021). While being able to perform neural induction without different media supplementation is a gold standard approach for proving the neurogenic potency of a system, it doesn’t account for changing nutrient requirements as cells adopt a neuronal fate. Further, the duration of induction is important. A one-day pulse of doxycycline in induced sensory neurons can produce a phenotypically and functionally different population than a 14-day pulse of dox (Nickolls et al., 2020). Though the exact reason why prolonged induction produced different cell fates is unknown, induction duration remains a crucial part of protocol optimization.

## Maturation and neural activity

Aside from morphology, one of the most common benchmarks for neural maturity is the ability to form functional synapses and display electrophysiological activity. While patch clamp electrophysiology remains a gold standard approach, microelectrode arrays (MEAs) are becoming an increasingly commonly used tool for measuring neural activity from hundreds or thousands of cells simultaneously (Novellino et al., 2011; Odawara et al., 2014; Mossink et al., 2021). Consisting of a grid of electrodes embedded in a cell culture surface, MEAs allow for a single plated population of neurons to be monitored over hours, days, or weeks without disturbance (Nehme et al., 2021). Temporal changes observed in neuronal firing and network formation, as measured on an MEA (e.g., network bursts, network synchrony, etc.), may also reflect neuronal maturation. Multi-well MEA formats even allow for scaled screening approaches with neural activity as a readout, to assess how experimental conditions (e.g., application of drugs/neurotransmitters, change in oxygen or carbon dioxide concentration, disease state, stimulation, etc.) within the MEA alter these characteristics of neuronal maturation.

While monitoring electrophysiological activity can give an assessment of maturity as neurons are kept in culture for increasing amounts of time, it does nothing to speed the maturation process. However, due to age being such a strong risk factor for many neurodegenerative diseases, there is an experimental need to obtain fully mature or even aged neuronal phenotypes for therapeutic screening. Many techniques exist to try to advance neural maturation for both directed and induced populations. One of the most common methods is to co-culture neurons with glia [e.g., primarily astrocytes (Tang et al., 2013; Odawara et al., 2014; Nehme et al., 2021)]. Other groups have transitioned neurons into media more permissible to synaptic activity (Satir et al., 2020), altered the extracellular matrix (Hyysalo et al., 2017), or cultured neurons with their target populations (Bubnys et al., 2019; Vila et al., 2021). Directly inducing activity in neurons, either through optogenetics or electrical stimulation, has also been shown to improve morphological and electrophysiological properties (Park et al., 2015; Latchoumane et al., 2018; Giraldo et al., 2020; Pagan-Diaz et al., 2020). Some compounds, such as the Notch inhibitor DAPT, have been shown to speed the rate of differentiation in neurons and shorten the timeline to synaptic activity, and ongoing studies are searching for other compounds to further reduce barriers to maturation (Borghese et al., 2010). Lastly, expression of progerin, a protein associated with premature aging in Hutchinson-Gilford progeria syndrome, induces many phenotypes of aging in both stem cells and neurons (Miller et al., 2013). No single maturation method will work for every experiment, and as with differentiation methods, a combinatorial approach may produce the most robust results.

## Delivery of inducible genetic elements

Various methods can be used to express induced elements, including viral integration, targeted safe-harbor integration, transposon integration, and even episomal expression. For purely research purposes, the method used to introduce an inducible cassette can be left up to researcher preference and familiarity. However, the safety and likelihood of integration of the delivery system is especially important if an engineered genetic system may move toward clinical trials. Retroviral and lentiviral transfections are highly efficient means of integrating genetic cassettes (Ma et al., 2003; Cao et al., 2010). Similarly, transposon-based piggybac systems can yield efficient integrations and stable expression, but with the additional benefit of transgene excision upon re-expression of the transposon (De Santis et al., 2018). Further, both transposon and donor DNA vectors can be introduced with simple electroporation, nucleofection, or lipofection, making them safer to work with than viruses.

Safe harbor loci, such as AASV1 or CLYBL, can be targeted using specific TALENs or CRISPR/Cas machinery. Functionally similar to the ROSA26 locus in mice, these regions allow for a site-specific integration without disrupting necessary genes, and have proven to be useful sites in which to integrate and standardize inducible gene systems since each clonal cell line will have a relatively uniform expression level, with one or two copies available.

One can express a genetic cassette from an episomal vector. Episomal vectors do not integrate into the genome and can maintain stable expression for extended periods of time. RNA-based Sendai viruses are commonly used to over-express Yamanaka reprogramming factors for iPSC production, but have also been used to express transcription factors to induce neurons (Goto et al., 2017). Plasmids containing the Epstein-Barr virus OriP/EBN1A features replicate within cells and are segregated with chromosomes during cell division, allowing long-term expansion of cell lines (Yates et al., 1985; Conese et al., 2004). Miniplasmids, lacking any bacterial sequences, are less likely to be silenced, but also lack replication competence and are diluted out in actively dividing cell populations. This limitation has been overcome by the production of pSMAR vectors, which contain scaffold/matrix attachment regions (SMARs) that allow them to be replicated and maintained throughout many mitotic cell divisions in both mouse and human stem cells and throughout differentiations (Mulia et al., 2021; Roig-Merino et al., 2022). Interestingly, while stable during mitotic division, pSMAR vectors are lost upon meiotic divisions and cannot be propagated through the germ line. Synthetic mRNA molecules can also be readily delivered into cells and maintain expression of transgenes long enough to induce cell fates from stem cells, but may require re-transfection for sustained expression (Warren et al., 2010; Goparaju et al., 2017; Azimi et al., 2018).

## Expanding the system and building in complexity

As outlined above, deep understanding of the *in vivo* development of a neuron population of interest can allow for genetic engineering techniques to be leveraged to efficiently generate large numbers of pure neurons for study, as has been shown with the induced MN system. However, the resulting control over cell identity also allows for heterogenous systems, such as those produced during directed differentiations and which better recapitulate the *in vivo* environment of the neuron, to be “built” with reliable and reproducible complexity. For instance, by combining two or more induced cell lines together in defined proportions, one could pre-determine the relative ratio of each cell type in the resulting neuronal culture. This would reduce the need for cell selection or dissociation and sorting, while maintaining long-term developmental contacts. Possessing control over cell proportions would allow researchers to ask targeted questions about how subtle shifts in the balance of excitatory and inhibitory populations influence circuit activity or disease phenotypes in ways that are difficult to achieve using purely directed differentiations.

Another potential application of engineered cells is to understand the progression and spread of disease. For instance, in ALS, MNs are known to specifically degenerate, though it remains undetermined whether the disease begins at the neuromuscular junction (NMJ) or in the motor regions of the cortex, termed the bottom-up and top-down models, respectively. Recent evidence also points to spinal interneurons being lost around the same time as MNs (Salamatina et al., 2020). Using innovative co-culture systems and induced cells that produce muscle, MNs, and spinal INs, one could mix not only the cell types, but also cell lines with disease associated genetic mutations (Andersen et al., 2020; Vila et al., 2021). Such a system could help elucidate which aspects of ALS disease progression are cell autonomous, and which result from compounded susceptibility throughout the circuit.

Induced cell identities can also add an orthogonal approach to a directed differentiation in order to obtain a fractional population of cells from a different CNS region or a difficult-to-generate cell type. For example, induced overexpression of SOX9 was shown to produce oligodendrocyte precursor cells (OPCs) from stem cells (Ng et al., 2021). By mixing SOX9-expressing cells with wild-type cells in a cortical organoid differentiation, organoids developed myelinating cells by day 30 instead of the 100 + days observed in wild-type only organoids. Thus, induced cell lines can help improve directed differentiation models by adding to the cellular complexity, not just simplifying it.

Of course, identity is not the only feature that can be engineered into cells. We now possess a wide array of genetically encoded tools to augment cell function, such as the channelrhodopsins, halorhodopsins, and designer receptors exclusively activated by designer drugs (DREADDs). These tools allow for modulation of neural activity in real time upon application of an exogenous stimulant in the form of light or specific ligands. By incorporating these tools into the systems outlined above to generate specific types of neurons, one can closely examine the contribution of these cell types to circuit activity or function. Modified neural activity can also be used to enhance integration or reintegration into functional circuits. For instance, increased neural activity has been reported to improve functional recovery in spinal dorsal roots, retina, motor cortex, and sciatic nerve, and the spinal cord itself (Carmel and Martin, 2014; Lim et al., 2016; Ward et al., 2016; Wu et al., 2020; English et al., 2021; Petersen et al., 2021). Incorporation of similar opto-or chemogenetic modulators into transplanted cells for therapies could be another way to promote cell survival, integration and plasticity, such as in phrenic and autonomic circuits after spinal cord injury (Zholudeva et al., 2018; Squair et al., 2021). Recently developed synthetic biology systems and modulations of cellular adhesion molecules have also been demonstrated to be able to control cell fate and spatial patterns (Libby et al., 2018, 2019; Toda et al., 2018, 2020). Further development of these systems and incorporation into specific neuron populations produced *in vitro* could allow researchers to control boundary formation between two populations of neurons or even direct their synaptic connections to create neuro-chip circuits.

## Concluding remarks

As highlighted in this review, advances in cellular engineering techniques have paved a new era of disease modeling, therapeutic development and a giant leap toward personalized medicine. Indeed, several new clinical trials are already underway to bring stem-cell derived neurons to patients. The development of specific neural cell types, co-cultures, and organoids will continue to greatly improve our understanding of neural development, network formation, and the processes that lead to change in neural injury and disease. Production of highly pure, relevant cell types at scale will be necessary to model circuits, use in high-throughput screens, and translate cell-replacement therapies to preclinical and clinical settings. Therefore, we posit that “hybrid approaches” that combine aspects of both directed and induced differentiation strategies will allow for the reproducible and flexible generation of desired cell types (Figure 2A). As discussed above, early regionalization steps are critical or refining the cell types produced by both directed and induced differentiation strategies and can be achieved by manipulating a limited number of developmental pathways. Subsequent induction of neurogenic or cell-type specific transcription factors, miRNA, or shRNA, along with exogenous small molecule supplementation will serve to further restrict cell fate to desired cell-types. Hybrid differentiation protocols will inevitably enhance our ability to create more specific neural components that can be used to develop treatments for neural injury and disease.

## Author contributions

NE and LZ: conceptualization. NE: original draft. All authors writing and editing, contributed to the article, and approved the submitted version.
